# The Efficacy of Ozone Prolotherapy Compared to Intra-Articular Hypertonic Saline Injection in Reducing Pain and Improving the Function of Patients with Knee Osteoarthritis: A Randomized Clinical Trial

**DOI:** 10.1155/2021/5579944

**Published:** 2021-08-03

**Authors:** Hamid reza Farpour, Alireza Ashraf, Seyed Saeed Hosseini

**Affiliations:** ^1^Shiraz Geriatric Research Center, Department of Physical Medicine and Rehabilitation, Shiraz University of Medical Sciences, Shiraz, Iran; ^2^Bone and Joint Diseases Research Center, Department of Physical Medicine and Rehabilitation, Shiraz University of Medical Sciences, Shiraz, Iran; ^3^Student Research Committee, Shiraz University of Medical Sciences, Shiraz, Iran

## Abstract

**Background:**

Knee osteoarthritis is a common disease that is associated with chronic pain and disability in patients. Prolotherapy is a complementary therapeutic approach for improving pain and function in patients with osteoarthritis. We aimed to compare the effect of hypertonic saline with ozone plus hypertonic saline in improving the symptoms of osteoarthritis in the patients. *Materials and Method*. In this clinical trial, thirty-four adults with painful primary knee osteoarthritis for at least three months were randomized to two groups: ozone plus hypertonic saline 5% and hypertonic saline 5% alone. Prolotherapy and thrice follow-up with two-week intervals were done. The outcome measures included Oxford Knee Scale (OKS), Western Ontario McMaster University Osteoarthritis Index (WOMAC), and Visual Analog Scale (VAS), which were obtained from the patients before the injection and after the 2^nd^ and 4^th^ weeks after the start of the study.

**Results:**

The mean age of the participants was 60.12 ± 7.54 years. There were no statistically significant differences between demographic characteristics before the injection between the two groups (*p* > 0.05). The results showed that VAS and OKS values decreased over time (*p* < 0.001) in each group, but there was no significant difference in the reduction of those between the two treatment groups (*p* = 0.734 and *p* = 0.734, respectively). Both interventions improved the mean values of WOMAC pain, WOMAC stiffness, WOMAC act, and WOMAC total. However, there was no significant difference in WOMAC pain reduction rate (*p* = 0.465), WOMAC stiffness rate (*p* = 0.656), WOMAC act rate (*p* = 0.376), and WOMAC total rate between the two methods (*p* = 0.528).

**Conclusion:**

The results showed that intra-articular prolozone therapy and hypertonic saline injection can lead to improvement of pain and function in patients with knee osteoarthritis at the same status without any significant difference.

## 1. Introduction

Osteoarthritis, the most common form of arthritis, is the leading cause of musculoskeletal pain and disability worldwide [1]. Osteoarthritis (OA) is an age-related degenerative disease resulting from articular cartilage failure induced by a complex interplay of genetic, metabolic, biochemical, and biomechanical factors with secondary components of inflammation leading to degradation of the cartilage, bone, and synovium [2, 3]. Its prevalence has been increasing given the increasing proportion of older people in the society [4].

In industrialized societies, OA is the leading cause of physical disability, increases in healthcare usage, and impaired quality of life [5]. The societal and personal burden of disease is high due to utilization of healthcare resources, time off work, and individual morbidity. There is no cure for the knee OA [5, 6]. However, several therapeutic options might be helpful in reducing symptoms, but they do very little in changing the biochemical environment or the degree of degeneration [3, 4].

Conservative treatment usually includes lifestyle modification, strengthening exercises and physical therapy, simple analgesics (nonsteroidal anti-inflammatory drugs), topical treatments [7, 8], different intra-articular injections, and supplements such as chondroitin sulphate and glucosamine [9, 10]. The current medical treatment strategies for OA aim at pain reduction and symptom control rather than disease modification [11]. These pharmaceutical treatments are limited and can have unwanted side effects [12].

Based on the multifactorial etiology of osteoarthritis and the pressing need to find new therapeutic options, recent evidence suggests that prolotherapy has a role in the routine care of osteoarthritis [13, 14]. Several studies reported that prolotherapy could be beneficial in the management of chronic tendinopathies and knee OA [13, 15].

Prolotherapy (prolo, an abbreviation of proliferation) is an injection-based therapy for chronic musculoskeletal pain conditions including osteoarthritis and overuse tendinopathy [16]. It has been termed a “regenerative” injection therapy due to these purported effects [15, 16]. One mechanism of prolotherapy is that the hyperosmolar solutions hyperpolarize the nerves by opening their potassium channels, thus decreasing transmission in the nociceptive pain fibers [17]. Additionally, hypertonic solutions are thought to produce an inflammatory response through the recruitment of chemical mediators and growth factors that stimulate local healing of injured extra- and intra-articular tissue. However, further definitive evidence regarding basic science remains in progress [16, 17].

Ozone (O_2_–O_3_) is an allotropic form of oxygen which has anti-inflammatory, analgesic, trophic, and immunomodulatory properties [18, 19]. Moreover, some newly reported studies have analyzed the effect of ozone-oxygen injection on joint and spine osteoarthritis and found that ozone had a significant painkiller effect on osteoarthritis of the joints and spine [20]. Also, its long-term effect on pain indicates the likelihood of some histological changes as the mechanism of its action [21]. Increased tissue oxygenation, painkiller, and anti-inflammatory effects through the antinociceptive device can be justified for the therapeutic effect of ozone in musculoskeletal patients [20–23]. Additionally, there are some intra-articular injections for patients who do not respond to routine conservative treatments and do not require arthroplasty simultaneously: injections such as hyaluronic acid (HA), corticosteroids, platelet-rich plasma (PRP), autologous blood, botulinum toxin (BTX), saline, and dextrose [24]. Although exact mechanism of prolotherapy is not clearly understood, but some trials support the theory of motivating an inflammatory cascade following cell shrinkage, which increase the release of collagen deposition and growth factors after injection of hypertonic saline [25, 26].

We conducted this trial to compare the efficacy of intra-articular injection of ozone hypertonic saline and hypertonic saline 5% in reducing pain and improving the function of patients with knee osteoarthritis.

## 2. Materials and Method

### 2.1. Study Population

This is a double-blind randomized clinical trial. Thirty-four patients who referred to Imam Reza and Shahid Rajaee Clinics affiliated to Shiraz University of Medical Sciences in March 2016 to March 2017 were selected. The inclusion criteria were having 1^st^ or 2^nd^ grade of OA based on radiologic Kellgren-Lawrence criteria [27] and having symptoms of knee osteoarthritis for at least three months after receiving paracetamol, NSAIDs, opioids, or physical and therapeutic exercises, with no other neuromuscular diseases.

Patients who had evidence of 3^rd^ or 4^th^ grade of OA based on radiologic Kellgren-Lawrence criteria; a history of knee surgery (or joint replacement), trauma, any intra-articular injection in the past 3 months, or prolotherapy in the past year; lower extremities deformity, any kind of disease disturbing the symptoms of knee pain such as active lumbar radiculopathy and neuropathy; and diabetes or rheumatic diseases such as rheumatoid arthritis, lupus, Ritter's disease, gout, and Brucella were excluded from our study. Moreover, during the study period, if patients had severe allergic reaction to the specified drug or had inability to cooperate in filling out forms (cognitive impairments or language problems), they were excluded.

In order to deal with possible side effects, the research team offers a solution for patients. In order to achieve this goal, in case of emergency, it is possible for the patient to go to the clinic in person. It also explains how people access the doctor and the research team by providing a landline number and cellphone to the patient.

### 2.2. Random Allocation and Blinding

**Figure 1:**
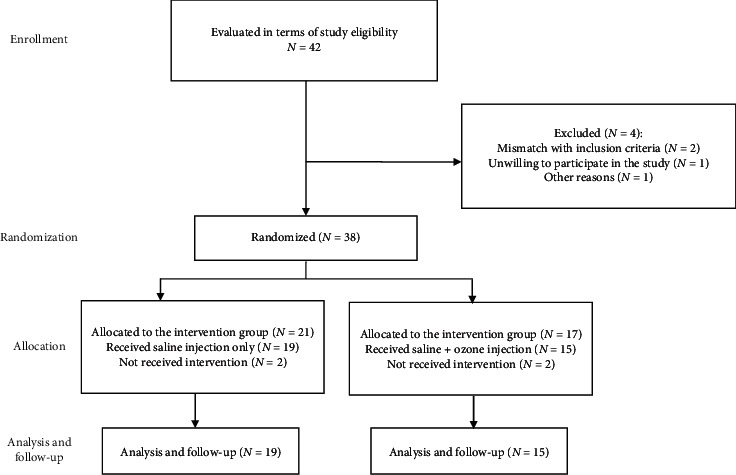
Consort flow chart of our clinical trial evaluating the efficacy of ozone prolotherapy compared to intra-articular hypertonic saline injection in reducing pain and improving the function of patients with knee osteoarthritis.

The sample size was determined by considering a significance level of 0.5, power of 0.80, and probable dropout rate of 20%. The sample size was approximated to be 21 patients in each group (Figure 1). We used a computer-based program with block randomization protocol, and the study investigators were blinded to the method of block size factor of four. Patients were assigned to two groups of study: knee-intra-articular injection of hypertonic saline alone and combination injection of hypertonic saline and ozone. Investigators who were evaluating the treatment were unaware of the allocation of treatment.

### 2.3. Intervention

The patients in group A received 4 ml saline 5% + 4 ml lidocaine 2% + 7 ml ozone at a concentration of 20 micrograms per liter in separate syringes, with intra-articular injection. The treatment in group B consisted of intra-articular injection of 4 ml saline 5% + 4 ml lidocaine 2%. Both groups were educated and recommended to follow the correct lifestyle and practice the proper exercises to reduce the knee pain, as explained in the clinic. For each of the two groups, three times of injection were performed at 2 weeks' interval. The patient was placed in a supine position, with knee flexion measuring 10 to 15°, and the landmark of injection area on the lateral side of knee was specified. Then, injection was done using needle G22 after aspiration and ensuring correct needle placement in the joint performed.

### 2.4. Data Collection

Both groups completed the standard questionnaire including Western Ontario and McMaster Universities Arthritis Index (WOMAC) [28], Oxford Knee Scale, (OKS) [29] and Visual Analog Scale (VAS) before and after treatment at weeks 2 and 4 after the last injection. The overall satisfaction from each procedure was asked after three weeks of intervention.

### 2.5. VAS Criteria

Pain severity was measured by a ten-point pain Visual Analog Scale (VAS).

### 2.6. WOMAC Standard Questionnaire

This questionnaire is a measure of performance that examines three categories of functional characteristics including pain (5 questions) and physical function (17 questions). Each question is rated as none (0), mild (1), moderate (2), severe (3), or very severe (4). If the patient scores less in this questionnaire, it means that she/he is in a better condition.

### 2.7. OKS Standard Questionnaire

The OKS provides a single summed score, which reflects the severity of problems that the respondent has with his/her knee. The questionnaire consists of 12 questions, each rated as none (4), mild (3), moderate (2), severe (1), or very severe (0). If the patient scores higher in this questionnaire, it means that she/he is in a better condition.

### 2.8. Statistical Analysis

The results of the present study were analyzed by descriptive (mean and standard deviation) and analytical tests (*t*-test, repeated measures analysis of variance, and chi-square), using SPSS version 19 software.

### 2.9. Ethical Considerations

The study method was approved by the Medial Ethics Committee of Shiraz University of Medical Sciences (SUMS) with the reference number “15497.” The aim of the study was to explain orally to all participants before they participated in the study. Also, the researcher obtained written informed consent. The enrollment of the patients initiated. The study protocol was registered as a clinical trial under registration ID: IRCT20180127038527N1 at the Iranian Registry of Clinical Trials (http://www.irct.ir); participation in the study was voluntary and the patients were free to leave the study at any time. Methods and design were not changed after we commenced the trial. If any complication occurred during the study, the researcher followed up and treated the patients completely. We tried to prevent any problem for the patients; we recommended exercise and lifestyle modification and prescribed acetaminophen if there was severe pain.

## 3. Results

Out of 42 patients assessed to enter the study, 38 were randomized to two groups. With omission of 2 patients in each group regarding personal reasons, we finally conducted the study on 34 patients, including 15 patients in the ozone + hypertonic saline group (male = 2, female = 12) and 19 patients in the hypertonic saline group (male = 4, female = 15). The mean age of the participants was 60.12 ± 7.54 years. There was no statistically significant difference between the saline + ozone and saline groups considering demographic data (Table 1, *p* > 0.05) and evaluation of VAS, OKS, and WOMAC (and subscales) questionnaires before injection (Table 2).

According to Table 3, the values of VAS reduced over time (*p* < 0.001) in each group independently, but comparison of the two groups showed no significant difference (*p* = = 0.57).

Indeed, the results showed that there was not any significant difference in the increase in the OKS value between the two intervention groups (*p* = 0.42). This means that the pattern of OKS changes over time does not differ significantly between the two groups.

As demonstrated in Table 4, the test results declared that the effect of time was significant and changed the average WOMAC pain values (*p* < 0.001). In the hypertonic saline + ozone and hypertonic saline groups, from the beginning until two weeks after the injection, WOMAC pain values increased slightly; then, it decreased from two weeks to four weeks after the intervention. However, in general, there was not any significant difference in WOMAC pain reduction between the two treatment methods (*p* = 0.46). Also, the test results showed that the effect of time was significant and changed the average WOMAC stiffness values (*p* < 0.001). There was not any significant difference in WOMAC stiffness reduction between the two treatment methods (*p* = 0.65).

About the results of the WOMAC function test revealed that the effect of time was also significant and changed the average WOMAC function values (*p* < 0.001) in each group. There was not any significant difference in WOMAC function reduction between the two intervention methods (*p* = 0.37). In the last three subscales, the results showed that the effect of time was significant and changed the average WOMAC total values (*p* < 0.001), but finally there was not any significant difference in WOMAC total reduction between the two intervention methods (*p* = 0.52).

## 4. Discussion

Osteoarthritis (OA) has a marked impact on the patients' quality of life and comorbid conditions as well as a dramatic economic cost around the world [4]. Therefore, offering directed treatments that are safe, cost effective, and beneficial to the patient will be of paramount importance [3, 10]. In the present study, we focused on intra-articular injection and its effectiveness. There are few studies in which prolotherapy such as ozone and hypertonic dextrose solution is used to manage knee OA, but the results about hypertonic saline effectiveness are not conclusive [30, 31]. We compared the two methods of intra-articular injection (hypertonic saline alone and combination of ozone and hypertonic saline) in patients with grade 1 and 2 osteoarthritis and finally concluded that two methods are clinically so useful in relieving the patients' pain and increasing functional abilities. Although there was not any significant difference between the two groups, both of them were found to be effective.

Intra-articular ozone-oxygen injections, particularly for the knee and shoulder joints, have shown themselves to be effective and relevant in acute and chronic painful diseases of the joints [20, 22]. However, there is little evidence for the effects of ozone on the symptoms of patients with knee osteoarthritis. In recent researches, several biological effects have been suggested for ozone, such as increased oxygen delivery to the tissues and sedative and anti-inflammatory effects and this ozone function could be a good justification for improving the symptoms in patients with osteoarthritis [32, 33].

Based on our results, we found that the mean pain score in terms of VAS in the ozone + hypertonic saline group did not make a significant difference in any of the intervention times compared to the hypersaline group. In the same line with our findings, Raeissadat et al. [34] compared the effects of ozone therapy versus hyaluronic acid (HA) intra-articular injection in knee OA patients; they found that both ozone 10 cc of ozone as an oxygen-ozone solution with the precise concentration of 30 *μ*g/mL was injected into the affected knee joint of patients in the ozone group and HA (20 mg/2 mL solution) could be effectively used for improving the function and reducing pain in selected knee OA patients, but neither of the two showed any superiority at 6-month follow-up. Ozone dosage for injection was more than that in our study in this survey. Also in another similar study, Momenzadeh et al. assessed the effect of two medical treatments using oxygen-ozone mixture (15 *μ*g/mL) and HA on pain and disability of patients with KOA. They concluded that the use of two interventions to relieve pain and disability is effective without any significant difference. Considering the mean dose of ozone use in Momenzadeh et al.'s study, we declare that ozone therapy in lesser amounts than in our study could be effective. [35].

Hashemi et al. [36] compared prolotherapy with hypertonic dextrose and ozone in patients with KOA. Their results showed that prolotherapy with dextrose (12.5%) and prolozone (15 g/mL) resulted in the same pain relief or functional improvement in patients with mild to moderate KOA. In another study, Feng et al. evaluated and compared intra-articular injection of ozone in the knee and taking celecoxib plus glucosamine orally in reducing pain. Pain intensity was decreased in the two groups, but this improvement in the ozone group was insignificantly faster [37]. They administered ozone in the same dose, but three times more than our study regarding the injected amounts. According to our intra-articular dosages used for patients, it seems that increase in the amount of ozone injections not only does not have more efficacy but also causes much more complications.

Our results also showed that in the study groups, the mean OKS score increased significantly during the follow-up in each group separately although the difference in this increase in the two groups was not significant.

Indeed, our findings showed that the mean amounts of all four WOMAC subscales increased from the beginning to two weeks after the intervention slightly and then decreased from two weeks to four weeks after the intervention. In Raeissadat et al.'s study, the total WOMAC score decreased significantly in the ozone group and in the HA group. A similar trend was observed in pain improvement according to WOMAC pain, stiffness, and function significantly improved in each groups independently, but no difference was found between the groups [34]. Similarly, in Momenzadeh et al.'s study, the number of WOMAC base at the O_2_-O_3_ group and HA group reduced in the first and second months after the last injection. It can be concluded that the use of pharmacological treatments is effective in reducing pain and disability caused by KOA, but the two treatments for oxygen-ozone mixture and HA worked similarly [35]. In line with our results, Fernández Cuadros et al. evaluated ozone therapy effects on the quality of life measured by the WOMAC-pain, WOMAC-stiffness, and WOMAC-function subscales in the KOA patients. All the subscales plus the WOMAC total score were decreased. They conclude that ozone is a safe medical treatment that can significantly improve pain, stiffness, and function in patients with KOA. The study showed a good level of evidence as well as a good grade of recommendation that allows us to consider ozone as a conservative therapeutic option in the treatment of KOA [38]. Also, in Lopes de Jesus et al.'s study, on pain intensity, it was observed that the results of WOMAC pain demonstrated a reduction in both groups (placebo and ozone groups) from baseline to the other follow-up times; however, the treated group presented a lower score than the placebo group. According to WOMAC, the parameter joint stiffness and physical activities also presented a significant difference with better results for the group treated with ozone [39]. However, in Hashemi et al.'s study, it was found that the average total WOMAC score increased after the intervention compared to before it, and this means that in their study, unlike the present study, no improvement was seen. It was also observed that both methods altered pain and function in patients similarly [36]. Dosage of injected ozone and follow-up time could be considered as causative factors of this disharmonic result.

In the present study, the results showed that hypertonic saline injection alone has an effective impact on VAS, OKS, and WOMAC scores of KOA patients. However, there are few studies which have indicated the effectiveness of hypertonic saline [30, 31].

Regarding the differences between our results and those of other studies, we can mention its limitations such as low sample size and short-term follow-up, while long-term follow-up seems necessary. It was also stated that there was no significant relationship between the patients' satisfaction and treatment groups, so the type of intervention did not affect the patients' satisfaction and no considerable adverse effect was seen in the participants; mild local pain was just observed in some patients in both groups who were relieved by nonsteroidal anti-inflammatory drugs. Indeed, using higher authorized doses of ozone, even 35, to reach the probable efficacy in OA patients is suggested.

## 5. Conclusion

The results of the present study showed that prolotherapy could improve pain and function in patients with osteoarthritis, but no significant difference in effectiveness was seen between the two types of treatment methods used in the present study. Given the fact that our study has been associated with limitations such as short-time follow-up, different results may be observed in other conditions. In addition, the same statistically significant results of the two methods would be due to the selected dose. Therefore, it is recommended that future studies should be done with a higher sample size and with more tracking times as well as an increase in ozone concentration and longer follow-up.

## Figures and Tables

**Table 1:** Demographic characteristics of the patients who participated in the intervention groups.

Groups	Ozone + hypertonic saline	Hypertonic saline	*p* value
Demographic factors	Mean ± SD	Mean ± SD	
Age (year)	57.5 ± 7.17	62.05 ± 7.4	0.08
Height (cm)	161.5 ± 10.08	158.47 ± 1.89	0.36
Weight (kg)	76.92 ± 8.89	72 ± 9.81	0.17
BMI	29.81 ± 5.22	28.48 ± 3.52	0.42

BMI, body mass index.

**Table 2:** Baseline VAS, OKS, and WOMAC scores of the intervention groups.

Groups	Hypertonic saline	Ozone + hypertonic saline	*p* value
Type of questionnaires	Mean ± SD	Mean ± SD	
VAS	7.6 ± 2	7.2 + 2.8	0.57
OKS	22.67 ± 9.46	20 ± 7.33	0.36
WOMAC			
Pain	10.94 ± 2.73	10.85 ± 3.34	0.93
Stiffness	4.05 ± 1.84	3.20 ± 1.72	0.62
Function	36.61 ± 8.69	37.21 ± 42.35	0.29
Total score	51.88 ± 9.88	51.28 ± 12.56	0.88

VAS, Visual Analog Scale; OKS, Oxford Knee Scale; WOMAC, Western Ontario McMaster University Osteoarthritis Index.

**Table 3:** Comparison of VAS and OKS in both groups.

Scale		Hypertonic saline group	Ozone + hypertonic saline group	*p* value within groups	*p* value between group
VAS (mean ± SD)	Baseline	7.6 ± 2	7.2 + 2.8	<0.001	0.73
	2^nd^ week	7.4 ± 2.29	7.4 ± 2.29		
	4^th^ week	5.47 ± 1.98	5.73 ± 2.21		

OKS	Baseline	22.67 ± 9.46	20 ± 7.33	<0.001	0.42
	2^nd^ week	20.13 ± 7.94	20.16 ± 7.56		
	4^th^ week	29.67 ± 10.64	26.26 ± 9.02		

VAS, Visual Analog Scale; OKS, Oxford Knee Scale.

**Table 4:** Comparison of WOMAC and subscales between the two groups.

Scale		Hypersaline group (mean ± SD)	Ozone + hypersaline group (mean ± SD)	*p* value within groups	*p* value between groups
WOMAC. pain	Baseline	10.94 ± 2.73	10.85 ± 3.34	<0.001	0.46
	2^nd^ week	11.73 ± 2.68	13.28 ± 4.84		
	4^th^ week	8.73 ± 3.01	9.42 ± 5.72		
WOMAC. stiffness	Baseline	4.05 ± 1.84	3.2 ± 1.72	<0.001	0.65
	2^nd^ week	4.21 ± 1.75	4.53 ± 2.09		
	4^th^ week	3.15 ± 1.06	3.2 ± 2.04		
WOMAC. function	Baseline	36.61 ± 8.69	37.21 ± 42.35	<0.001	0.37
	2^nd^ week	37.88 ± 11.38	42.35 ± 15.09		
	4^th^ week	28.94 ± 11.44	31.21 ± 17.95		
WOMAC. total	Baseline	51.88 ± 9.88	51.28 ± 12.76	<0.001	0.52
	2^nd^ week	53.83 ± 14.22	60.14 ± 21.42		
	4^th^ week	41.16 ± 11.44	43.92 ± 25.4		

WOMAC, Western Ontario McMaster University Osteoarthritis Index.

## Data Availability

The data have been recorded by the authors and are available from the corresponding author.

## References

[1] Reginster J. Y. (2002). The prevalence and burden of arthritis. *Rheumatology*.

[2] Samvelyan H. J., Hughes D., Stevens C., Staines K. A. (2020). Models of osteoarthritis: relevance and new insights. *Calcified Tissue International*.

[3] Abramoff B., Caldera F. E. (2020). Osteoarthritis: pathology, diagnosis, and treatment options. *Medical Clinics of North America*.

[4] Kloppenburg M., Berenbaum F. (2020). Osteoarthritis year in review 2019: epidemiology and therapy. *Osteoarthritis and Cartilage*.

[5] Briani R. V., Ferreira A. S., Pazzinatto M. F., Pappas E., De Oliveira Silva D., Azevedo F. M. d. (2018). What interventions can improve quality of life or psychosocial factors of individuals with knee osteoarthritis? A systematic review with meta-analysis of primary outcomes from randomised controlled trials. *British Journal of Sports Medicine*.

[6] Xie F., Kovic B., Jin X. (2016). Economic and humanistic burden of osteoarthritis: a systematic review of large sample studies. *Pharmacoeconomics*.

[7] Shoara R., Hashempur M. H., Ashraf A., Salehi A., Dehshahri S., Habibagahi Z. (2015). Efficacy and safety of topical Matricaria chamomilla L. (chamomile) oil for knee osteoarthritis: a randomized controlled clinical trial. *Complementary Therapies in Clinical Practice*.

[8] Farpour H. R., Estakhri F., Zakeri M., Parvin R. (2020). Efficacy of piroxicam mesotherapy in treatment of knee osteoarthritis: a randomized clinical trial. *Evidence-Based Complementary and Alternative Medicine*.

[9] Zampogna B., Papalia R., Papalia G. F. (2020). The role of physical activity as conservative treatment for hip and knee osteoarthritis in older people: a systematic review and meta-analysis. *Journal of Clinical Medicine*.

[10] Bert J. M., Endres N. K., Tucker C. J., Davey A. P. (2018). The conservative treatment of osteoarthritis of the knee. *Orthopedics*.

[11] Kan H. S., Chan P. K., Chiu K. Y. (2019). Non-surgical treatment of knee osteoarthritis. *Hong Kong Medical Journal*.

[12] Majeed M. H., Sherazi S. A. A., Bacon D., Bajwa Z. H. (2018). Pharmacological treatment of pain in osteoarthritis: a descriptive review. *Current Rheumatology Reports*.

[13] Hassan F., Trebinjac S., Murrell W. D., Maffulli N. (2017). The effectiveness of prolotherapy in treating knee osteoarthritis in adults: a systematic review. *British Medical Bulletin*.

[14] Farpour H. R., Fereydooni F. (2017). Comparative effectiveness of intra-articular prolotherapy versus peri-articular prolotherapy on pain reduction and improving function in patients with knee osteoarthritis: a randomized clinical trial. *Electronic Physician*.

[15] Kim S. R., Stitik T. P., Foye P. M., Greenwald B. D., Campagnolo D. I. (2004). Critical review of prolotherapy for osteoarthritis, low back pain, and other musculoskeletal conditions. *American Journal of Physical Medicine & Rehabilitation*.

[16] Siadat A. H., Isseroff R. R. (2019). Prolotherapy: potential for the treatment of chronic wounds?. *Advances in Wound Care*.

[17] Distel L. M., Best T. M. (2011). Prolotherapy: a clinical review of its role in treating chronic musculoskeletal pain. *PM&R*.

[18] Zanardi I., Borrelli E., Valacchi G., Travagli V., Bocci V. (2016). Ozone: a multifaceted molecule with unexpected therapeutic activity. *Current Medicinal Chemistry*.

[19] Bocci V. A. (2006). Scientific and medical aspects of ozone therapy. State of the art. *Archives of Medical Research*.

[20] Al-Jaziri A. A., Mahmoodi S. M. (2008). Painkilling effect of ozone-oxygen injection on spine and joint osteoarthritis. *Saudi Medical Journal*.

[21] Manoto S. L., Maepa M. J., Motaung S. K. (2018). Medical ozone therapy as a potential treatment modality for regeneration of damaged articular cartilage in osteoarthritis. *Saudi Journal of Biological Sciences*.

[22] Sconza C., Respizzi S., Virelli L. (2020). Oxygen-ozone therapy for the treatment of knee osteoarthritis: a systematic review of randomized controlled trials. *Arthroscopy: The Journal of Arthroscopic & Related Surgery*.

[23] Mishra S. K., Pramanik R., Das P. (2011). Role of intra-articular ozone in osteo-arthritis of knee for functional and symptomatic improvement. *Indian Journal of Physical Medicine and Rehabilitation*.

[24] Najafi S., Sanati E., Khademi M., Abdorrazaghi F., Mofrad R. K., Rezasoltani Z. (2019). Intra-articular botulinum toxin type A for treatment of knee osteoarthritis: clinical trial. *Toxicon*.

[25] Freeman J. W., Empson Y. M., Ekwueme E. C., Paynter D. M., Brolinson P. G. (2011). Effect of prolotherapy on cellular proliferation and collagen deposition in MC3T3-E1 and patellar tendon fibroblast populations. *Translational Research*.

[26] Rezasoltani Z., Taheri M., Kazempour Mofrad M., Mohajerani S. (2017). Periarticular dextrose prolotherapy instead of intra-articular injection for pain and functional improvement in knee osteoarthritis. *Journal of Pain Research*.

[27] Kohn M. D., Sassoon A. A., Fernando N. D. (2016). Classifications in brief: Kellgren-Lawrence classification of osteoarthritis. *Clinical Orthopaedics & Related Research*.

[28] Eftekhar-Sadat B., Niknejad-Hosseyni S. H., Babaei-Ghazani A., Toopchizadeh V., Sadeghi H. (2015). Reliability and validity of Persian version of Western Ontario and McMaster Universities Osteoarthritis index in knee osteoarthritis. *Journal of Analytical Research in Clinical Medicine*.

[29] Ebrahimzadeh M. H., Makhmalbaf H., Birjandinejad A., Soltani-Moghaddas S. H. (2014). Cross-cultural adaptation and validation of the Persian version of the oxford knee score in patients with knee osteoarthritis. *Iranian Journal of Medical Sciences*.

[30] Yelland M. J., Glasziou P. P., Bogduk N., Schluter P. J., McKernon M. (2004). Prolotherapy injections, saline injections, and exercises for chronic low-back pain: a randomized trial. *Spine*.

[31] Tavana B., Azizi S., Najafi S., Taftian E., Maghbouli N. (2019). The effectiveness of intra-articular injection of hypertonic saline in pain control and function of patients with knee osteoarthritis. *Journal of Orthopedic and Spine Trauma*.

[32] de Sire A., Stagno D., Minetto M. A., Cisari C., Baricich A., Invernizzi M. (2020). Long-term effects of intra-articular oxygen-ozone therapy versus hyaluronic acid in older people affected by knee osteoarthritis: a randomized single-blind extension study. *Journal of Back and Musculoskeletal Rehabilitation*.

[33] Oliviero A., Giordano L., Maffulli N. (2019). The temporal effect of intra-articular ozone injections on pain in knee osteoarthritis. *British Medical Bulletin*.

[34] Raeissadat S. A., Rayegani S. M., Forogh B., Hassan Abadi P., Moridnia M., Rahimi-Dehgolan S. (2018). Intra-articular ozone or hyaluronic acid injection: which one is superior in patients with knee osteoarthritis? A 6-month randomized clinical trial. *Journal of Pain Research*.

[35] Momenzadeh S., Poorfarrokh M., Hashemi M., Barikani A. (2014). Comparison of intra-articular oxygen-ozone and hyaluronic acid prolotherapy on pain and disability of osteoarthritis patients. *Research in Medicine*.

[36] Hashemi M., Jalili P., Mennati S. (2015). The effects of prolotherapy with hypertonic dextrose versus prolozone (intraarticular ozone) in patients with knee osteoarthritis. *Anesthesiology and Pain Medicine*.

[37] Feng X., Beiping L. (2017). Therapeutic efficacy of ozone injection into the knee for the osteoarthritis patient along with oral celecoxib and glucosamine. *Journal of Clinical and Diagnostic Research*.

[38] Cuadros M. E. F., Moro O. S. P., Florin M. J. A., Canelo J. A. M. (2017). Ozone improves pain, function and quality of life in patients with knee osteoarthritis: a prospective quasi-experimental before-after study. *Middle East Journal of Rehabilitation and Health*.

[39] de Jesus C. C. L., dos Santos F. C., de Jesus L. M. O. B., Monteiro I. (2017). Comparison between intra-articular ozone and placebo in the treatment of knee osteoarthritis: a randomized, double-blinded, placebo-controlled study. *PLoS One*.

